# Evaluation of early-phase ^18^F-Florbetaben PET as a surrogate biomarker of neurodegeneration: In-depth comparison with ^18^F-FDG PET at group and single patient level

**DOI:** 10.1177/13872877251340380

**Published:** 2025-05-08

**Authors:** Jose Antonio Lojo-Ramírez, Paula Fernández-Rodríguez, Miriam Guerra-Gómez, Alba Marta Marín-Cabañas, Emilio Franco-Macías, Jose Manuel Jiménez-Hoyuela-García, David García-Solís

**Affiliations:** 1 Instituto de Biomedicina de Sevilla, IBiS/Hospital Universitario Virgen del Rocío/CSIC/Universidad de Sevilla, España; 2 Servicio de Medicina Nuclear, Hospital Universitario Virgen del Rocío, Sevilla, España; 3 Unidad de Memoria, Servicio de Neurología, Hospital Universitario Virgen del Rocío, Sevilla, España

**Keywords:** Alzheimer's disease, dementia, amyloid PET, brain perfusion, Florbetaben, neuroimaging

## Abstract

**Background:**

Imaging biomarkers are essential in Alzheimer's disease (AD) diagnosis, particularly since the introduction of the ATN criteria by the NIA-AA. These criteria include amyloid-β plaques (amyloid PET), fibrillar tau (tau PET), and neurodegeneration (FDG PET or MRI). Early-phase amyloid PET imaging has shown a strong correlation with FDG PET at the group level.

**Objective:**

This study evaluates the comparability of early-phase FBB PET (eFBB) perfusion imaging and FDG PET metabolic imaging at both group and individual levels.

**Methods:**

A retrospective study included 103 patients with mild cognitive impairment (MCI) or mild dementia suspected of AD who underwent FDG PET and dual-phase ^18^F-Florbetaben PET (including a 5-min eFBB scan) between 2019 and 2023, along with 33 healthy controls. Imaging analyses included qualitative, semi-quantitative, and voxel-wise techniques to compare eFBB and FDG PET scans.

**Results:**

eFBB and FDG PET SUVR values showed a strong correlation across all brain regions (rho = 0.879). Visual assessments of eFBB and FDG PET by two raters achieved intra-observer agreement rates of 87.5% and 86.4%, respectively. Voxel-wise analysis revealed moderate to good overlap, as indicated by Dice-Sørensen coefficients, in the MCI and mild dementia groups. Discriminative performance between eFBB and FDG PET was comparable, with no significant differences as eFBB reliably reflected brain metabolic patterns observed on FDG PET, supporting its diagnostic utility.

**Conclusions:**

eFBB PET could serve as a surrogate biomarker for FDG PET in the diagnostic evaluation of neurodegenerative dementias. Dual-phase amyloid PET enables simultaneous assessment of neurodegeneration and amyloid burden, offering a streamlined and resource-efficient approach for clinical practice.

## Introduction

In recent years, imaging biomarkers have become a cornerstone in the diagnosis of Alzheimer's disease (AD), particularly following the publication of the ATN diagnostic criteria by the NIA-AA. Originally developed for research purposes, these criteria introduced three key imaging biomarkers for AD diagnosis: amyloid-β plaques (A) assessed through amyloid positron emission tomography (PET), fibrillar tau (T) evaluated via tau PET, and neurodegeneration (N) detected using ^18^F-fluorodeoxyglucose (FDG) PET or magnetic resonance imaging (MRI).^1^

More recently, the criteria have been updated to incorporate additional biomarkers, including novel blood-based biomarkers and imaging biomarkers for non-AD co-pathologies, such as white matter hyperintensities on CT or MRI. This shift moves AD diagnosis from being solely based on clinical presentation to one that integrates biological characterization.^2^

The pathophysiological process of AD is complex and is characterized by the abnormal processing of amyloid-β protein precursor, leading to the formation of amyloidogenic Aβ peptides that aggregate into neuritic plaques, and the presence of intracellular neurofibrillary tangles composed of a hyperphosphorylated form of the microtubule-associated protein tau, which contribute to neurodegeneration and cognitive decline.^3–7^

Currently, the definitive diagnosis of AD can be established not only through postmortem examination but also via in vivo biomarkers. Among these, amyloid PET imaging allows the in vivo assessment of Aβ plaque deposition using validated Aβ- binding tracers, such ^11^C-Pittsburgh compound-B (PiB), ^18^F-Florbetapir (FBP), ^18^F-Flutemetamol (FMM), and ^18^F-Florbetaben (FBB).^8–11^

In contrast, FDG PET is widely used to evaluate brain metabolism and neurodegeneration, as neuronal impairment leads to reduced brain metabolism, which FDG PET effectively captures. Similarly, cerebral blood flow (CBF) is considered an important biomarker in the study of various neurological disorders, including neurodegenerative and cerebrovascular diseases.^12–14^

Brain perfusion SPECT, despite being one of the most accessible techniques for assessing CBF, has limitations related to resolution and tracer stability.^15^ In comparison, ^15^O-Water PET, considered the gold standard for quantitative CBF measurements, is largely restricted to research centers with on-site cyclotrons due to the tracer's short half-life (2 min).^16^

Interestingly, the high lipophilicity of amyloid PET tracers enables early-phase cerebral perfusion imaging, yielding results comparable to those of ^15^O-Water PET. This dual-phase imaging protocol, which acquires perfusion images in the early phase and amyloid burden images in the late phase, offers significant resource and time savings.^17,18^

Early-phase amyloid PET imaging has demonstrated a strong correlation with FDG PET uptake at the group level, suggesting its potential as a biomarker for neuronal dysfunction.^19–34^ However, the number of studies evaluating its application at the single-patient level is extremely limited, underscoring the need for further exploration in this area.^35^ Although the available results are promising, additional research is required to validate its clinical utility in this context.

This study aimed to investigate the comparability between early-phase cerebral perfusion images obtained with FBB PET (eFBB) and cerebral metabolism images derived from FDG PET. Specifically, we analyzed these imaging modalities at both the group level, through qualitative and semi-quantitative analyses, and at the single-patient level, using voxel-wise mapping. Furthermore, we explored the potential clinical applicability of eFBB as a dual-phase imaging biomarker, leveraging its correlation with FDG PET to improve diagnostic accuracy and optimize resource utilization in routine practice.

## Methods

### Patients

This is a single-center observational retrospective study, which was approved by the local ethics committee conducted in accordance with the principles of the Declaration of Helsinki and the International Conference on Harmonization Good Clinical Practice. Informed consent was not required.

Our study included 103 patients who were attended at the outpatient Memory Clinic of a tertiary hospital, with MCI or mild dementia (dementia), suspected of AD. All of them underwent a dual phase amyloid-PET scan (early-phase [eFBB] and late-phase [lFBB]) with FBB, and a FDG PET scan, both between June 2019 and May 2023. The maximum time elapsed between scans was 12 months.

Additionally, a group of 33 healthy controls (HC) who underwent FDG PET at the Coma Science Group (University Hospital of Liege) was included to provide a reference group for voxel-wise analysis for both eFBB PET and FDG PET. The data includes subjects from a broad age range (19–70). FDG PET was performed about 30-60 min after intravenous injection of 150 or 300 MBq of FDG using a Gemini TF PET-CT scanner (Philips Medical Systems). A low-dose CT was acquired for attenuation correction, followed by a 12-min emission scan. The studies were reconstructed using a LOR-OSEM algorithm and reconstructed images contained 89 to 90 slices with 2 mm thickness, each containing 128 × 128 pixels of 2 × 2 mm (FOV 25.6 mm). The images of each subject were manually reoriented to the orientation of the MNI 152 1 mm3 template and were normalized to a 2 × 2 × 2 mm voxels FDG PET template and smoothed using a Gaussian filter with 14 mm FWHM. These images were obtained from the EBRAINS Research Infrastructure (https://www.ebrains.eu/).^36^

Demographic information and neurological exam data were collected from medical records. Neurological and neuropsychological exams included Mini-Mental State Examination (MMSE) total scores to measure global cognition, the TMA-93 for evaluating memory, and the GDS to get an overview of the stages of cognitive function.^37–41^

### Image acquisition

FDG PET and dual phase FBB PET were performed at the Department of Nuclear Medicine using a Discovery MI 5R digital PET/CT (GE Healthcare, US).

### FDG PET image acquisition

FDG PET was performed according to the European Association of Nuclear Medicine guidelines.^42^ All patients fasted for at least 4 h, and blood glucose levels were assessed before radiopharmaceutical injection. Blood glucose level was less than 150 mg/dL in all subjects. Patients were injected approximately 185 MBq of FDG via a venous cannula, and they were advised to rest for 30 min in a separate room with lights off and low noise level before undergoing FDG PET acquisition. After rest, a static emission frame was performed, lasting 15 min. Low-dose CT was performed before static acquisition, and these CT scans were used for attenuation correction. PET data were reconstructed using a 256 × 256 matrix, a Bayesian penalized-likelihood Q.Clear^®^ reconstruction algorithm and a slice thickness of 2.79 mm.^43^

### FBB PET image acquisition

*Early-phase image.* A dynamic emission recording was started immediately after radiopharmaceutical injection via venous cannula. Patients were injected with a standard dose of 296 MBq (± 10%) of FBB (Neuraceq^®^). The scan lasted 5 min (0–5 min post-injection). Low-dose CT was performed before dynamic acquisition, and these CT scans were used for attenuation correction. PET data were reconstructed using a 256 × 256 matrix, a Bayesian penalized-likelihood Q.Clear^®^ reconstruction algorithm, and a slice thickness of 2.79 mm (same algorithm implemented for PET FDG scans).

*Late-phase image.* A static emission recording that lasted 15–20 min was performed after 90 min from the FBB injection. Low-dose CT was performed before static acquisition, and these CT scans were used for attenuation correction. PET images were reconstructed using a 256 × 256 matrix, a Bayesian penalized-likelihood Q.Clear^®^ reconstruction algorithm (Discovery MI), and a slice thickness of 2.79 mm.

### Visual analysis of FDG PET and early-phase amyloid PET

For the interpretation of both FDG PET and eFBB PET images, three dimensional stereotactic surface projections (3D-SSP) were generated using the neuroimaging tool CortexID Suite (GE Healthcare, US), applying the FDG processing to both.^44,45^

Two independent Nuclear Medicine experts visually assessed the 3D-SSP images, using tracer uptake with global mean (GLM) scaling as a reference for both eFBB and FDG PET.

All readers were blinded to any identifying and clinical information. Four-item judgment was performed for the most likely of the following diagnoses (0 = Normal, 1 = Possible AD, = Probable AD, 3 = Others (FTLD, vascular…) on both PET images, separately. An agreement analysis of both scans was conducted for each reader (intra-observer), as well as an interobserver agreement analysis for each of the scans.

“Probable AD” was considered when a significant decrease in regional brain glucose metabolism affecting mainly the posterior temporoparietal, posterior and inferior temporal, and precuneus and posterior cingulate cortices, unilaterally or bilaterally.^45–51^ “Possible AD” when it only met the criteria for probable Alzheimer's disease but only partially or with a not very significant degree of hypometabolism.

We considered the remaining patterns of hypometabolism as “non-AD patterns,” according to the different findings in the literature described for other neurodegenerative diseases rather than AD, and no-degenerative pathologies (as FTLD, small vessel vascular pathology, etc.).^51–54^

### Semiquantitative analysis of FDG PET and eFBB PET

*Cortical surface VOI analysis.* Standardized uptake values (SUV) were obtained for each volume of interest (VOI). GLM was used as reference region for activity normalization. The SUV of each target region was divided by the SUV of the reference region, resulting in regional SUV ratios (SUVR) for both FDG PET and eFBB PET. The software package used to perform these analyses was Cortex ID. The composite value was defined as the arithmetic mean of the values of all target regions in both hemispheres.^55^ SUVR were generated automatically by CortexID for the following regions of both hemispheres: the prefrontal lateral cortex, prefrontal medial cortex, anterior cingulate cortex, posterior cingulate cortex, sensorimotor cortex, precuneus, parietal superior cortex, parietal inferior cortex, temporal lateral cortex, temporal mesial cortex, occipital lateral cortex, primary visual cortex. These VOIs were defined according to the ICBM152 atlas.^56^

*Semi-quantitative of late-phase Amyloid PET.* Amyloid PET scans were categorized as positive (Aβ+) or negative (Aβ−) based on the Centiloid (CL) scale, using a threshold of ≥ 35 CL (equivalent to SUVR = 1.27). The selection of this cutoff is based on prior validation studies demonstrating that CL values of 25–30 represent the upper limit of amyloid negativity, while CL ≥ 35 corresponds to a moderate-to-high amyloid burden confirmed by postmortem analyses.^57–61^ This criterion has been widely adopted in both research and clinical settings to ensure standardization across different tracers and robust differentiation between amyloid-negative and amyloid-positive individuals.^62^ Accordingly, we used CL ≥ 35 to dichotomize cases into AD and non-AD groups in subsequent analyses, including receiver operating characteristic (ROC) curve evaluations.

To determine the CL value, each subject's amyloid-PET image was spatially normalized to a total-FBB template and smoothed by the validated “robust PET-only Processing” (rPOP) method developed by Iaccarino et al. using Statistical Parametric Mapping software 12 (SPM, http://www.fil.ion/uc.ac.uk/spm) running on MATLAB (MathWorks 2020b) and Analysis of Functional Neuroimages (AFNI, https://afni.nimh.nih.gov/).^63^ Then, the neocortical standardized uptake value ratios (SUVRs) were quantified using the reference regions-of-interest (ROIs) established by the standard Global Alzheimeŕs Association Interactive Network (GAAIN, http://www.gaain.org/centiloid-project): the Global Cortical Target Region Subject Set (CTX)/ Whole cerebellum (WC), using the FMRIB Software Library v6.0 (FSL) statistics tools. Finally, we convert the SUVR to the CL using the formula specifically applicable to FBB developed by Rowe et al. but adapted to the rPOP processing method.^58^

### Single-subject voxel-wise analysis and ad meta–region of interest (meta-ROI) analysis

According to a validated SPM single-subject procedure, each PET images (FDG and eFBB) was analyzed for relative hypometabolism/ hypoperfusion using a 2-sample t-test compared the FDG PET images of the HC group. The statistical threshold for the resulting hypometabolic and/or hypoperfusion SPM maps was set at a p value of 0.05, uncorrected for multiple comparisons, considering significant clusters containing more than 100 voxels.^64^ The SPM maps were then binarized for further Dice-Sørensen (Dice) analyses, as a measure of concordance. Dice-Sørensen coefficient for binary maps A and B is defined as:

Dice=(2×|A∩B|)/(|A|+|B|).

It takes the value of 1 if A and B assume the same logical value in every pixel (high concordance), and a value of 0 if they always disagree (null concordance). It is interpreted as follows: <0.2, poor; 0.2–0.4, fair; 0.4–0.6, moderate; 0.6–0.8, good; and >0.8, excellent agreement.^65^ Additionally, the results were subclassified based on whether the PET amyloid scans were positive or negative, and whether the initial diagnosis was MCI or dementia.

Furthermore, SUVRs were computed for an AD specific meta-ROI previously described by Landau et al., which includes regions highly susceptible to neurodegeneration in AD.^66^ SUVRs were calculated as follows with FDG PET and eFBB PET normalized to the cerebellar cortex.

SUVR values were extracted for both, patients with amyloid-positive AD and HC, enabling a direct comparison between FDG PET and eFBB PET.

### Statistical analyses

All statistical tests were performed using the software IBM SPSS^®^ Statistics version 24 and the open statistical software Jamovi 2.5.4.^67^ For quantitative variables, the normal distribution was assessed with Shapiro-Wilk test and Kolmogorov-Smirnov test. Group correlations of regional SUVRs between eFBB and FDG images were evaluated using Spearman's rank correlation coefficient (rho).

To assess whether amyloid status influences eFBB results, rho were also calculated separately for amyloid-positive (Aβ+) and amyloid-negative (Aβ−) patients across all brain regions. A Fisher's z-transformation test was performed to statistically compare the correlation coefficients between Aβ+ and Aβ− groups. This test evaluates whether two independent correlation coefficients significantly differ from each other. A p-value < 0.05 was considered statistically significant.

For determining the most likely PET diagnosis, intra-reader agreement between eFBB and FDG, and inter-reader agreement for each of the scans were calculated using Cohen's Kappa. The Student's t-test and its non-parametric alternative Mann-Whitney U test were used to explore the mean differences between groups. Significant differences were determined to exist when p < 0.05. The Wilcoxon signed-rank test was used to evaluate the uptake difference between the values of FDG PET and eFBB PET.

Dice-Sørensen coefficients were calculated using MATLAB software (MathWorks 2020b), to quantify the whole-brain spatial overlap between hypometabolic and hypoperfusion binary maps at the single-subject level.

Finally, ROC curve analyses were conducted using amyloid PET (CL ≥ 35) as the gold standard for defining AD versus non-AD. Based on this criterion, the discriminative performance of FDG PET and eFBB was evaluated, highlighting the concordance and discordance between these imaging modalities in relation to the amyloid PET standard. The resulting areas under the curve (AUC) for both scans were compared using a DeLong test for 2 correlated ROC curves, setting the threshold for significance at a p < 0.05.^68^

## Results

### Participants demographic

Patient characteristics are detailed in Table 1. Of the 103 study patients, 45 (43.7%) were male and the mean age was 69.3 years (standard deviation [SD] = 7.52). In the Memory Clinic, the MMSE and TMA-93 were applied to all patients, with an average score of 22.55 (SD = 4.45) y 16.45 (SD = 8.05), respectively. Amyloid PET classification results, based exclusively on CL, identified 59/103 patients (57.3%) as Aβ+ (CL ≥ 35) and 44/103 (42.7%) as Aβ- (CL < 35). This classification based on CL served as the definitive criterion for all subsequent analyses. The diagnostic categorization of Aβ+ patients showed consistency across clinical subgroups, with 34/59 classified as dementia and 25/59 as MCI. The average time elapsed between FDG PET and FBB PET was 5.70 months (SD = 4.15).

**Table 1.** Demographic characteristic of patients.

Characteristic	Sample
n	103
Age	69.34 ± 7.52
Sex	
Male	45 (43.7%)
Female	58 (56.3%)
MMSE	22.55 ± 4.45
TMA93	16.45 ± 8.05
GDS	3.49 ± 0.68
Clinical status	
MCI	44 (42.7%)
Dementia	59 (57.3%)
Amyloid status	
Negative (CL < 35)	44 (42.7%)
Positive (CL ≥ 35)	59 (57.3%)
Clinical groups	
Aβ- MCI	19 (18.4%)
Aβ+ MCI	25 (24.3%)
Aβ- Dem	25 (24.3%)
Aβ+ Dem	34 (33.0%)

MMSE: Mini-Mental Status Exam; TMA93: Memory Associative Test of the district of Seine-Saint-Denis; GDS: Global Deterioration Scale; CL: Centiloid; MCI: mild cognitive impairment; Dem: Dementia.

### Correlation between eFBB and FDG PET SUVRs by cortical surface VOIs

Subregional SUVR and correlation coefficients for all cortical VOIs are summarized in Table 2. All FDG PET values in the whole brain, except for the left primary visual cortex, were significantly higher than those of eFBB PET. Likewise, statistically significant differences were observed in all cortical subregions except in the bilateral primary visual cortex and the bilateral lateral occipital cortex (p < 0.05; Wilcoxon signed-rank test).

**Table 2.** Subregional SUVRs, Spearman's rank correlation coefficients and Wilcoxon signed-rank test determined by comparing between regional eFBB and FDG PET with global mean (GLM) normalization. IQR: InterQuartile Range.

Brain region	eFBB (Median/IQR)	FDG (Median/IQR)	Correlation (rho)	p
R Hemisphere	0.90 ± 0.04	0.93 ± 0.05	0.545	<0.001
L Hemisphere	0.91 ± 0.04	0.92 ± 0.05	0.610	<0.001
Total Cortex	0.90 ± 0.04	0.92 ± 0.04	0.494	<0.001
R Lateral Prefrontal	0.96 ± 0.07	0.98 ± 0.07	0.570	<0.001
L Lateral Prefrontal	0.96 ± 0.06	0.97 ± 0.8	0.579	<0.001
R Medial Prefrontal	0.86 ± 0.09	0.88 ± 0.12	0.706	<0.001
L Medial Prefrontal	0.87 ± 0.09	0.89 ± 0.10	0.712	<0.001
R Sensorimotor	0.95 ± 0.08	0.99 ± 0.08	0.646	<0.001
L Sensorimotor	0.96 ± 0.08	0.99 ± 0.08	0.758	<0.001
R Anterior Cingulate	0.77 ± 0.12	0.79 ± 0.16	0.857	<0.001
L Anterior Cingulate	0.78 ± 0.13	0.79 ± 0.11	0.817	<0.001
R Posterior Cingulate	0.95 ± 0.12	0.98 ± 0.16	0.764	<0.001
L Posterior Cingulate	0.95 ± 0.13	0.98 ± 0.14	0.737	<0.001
R Precuneus	0.96 ± 0.09	1.00 ± 0.10	0.592	<0.001
L Precuneus	0.96 ± 0.08	1.00 ± 0.10	0.594	<0.001
R Superior Parietal	0.88 ± 0.12	0.90 ± 0.11	0.805	<0.001
L Superior Parietal	0.90 ± 0.11	0.92 ± 0.11	0.825	<0.001
R Inferior Parietal	0.94 ± 0.07	0.95 ± 0.06	0.694	<0.001
L Inferior Parietal	0.92 ± 0.08	0.93 ± 0.08	0.813	<0.001
R Lateral Occipital	1.01 ± 0.09	1.01 ± 0.10	0.762	<0.001
L Lateral Occipital	1.02 ± 0.09	1.02 ± 0.08	0.713	<0.001
R Primary Visual	1.08 ± 0.10	1.08 ± 0.15	0.619	<0.001
L Primary Visual	1.11 ± 0.19	1.09 ± 0.13	0.586	<0.001
R Lateral Temporal	0.87 ± 0.06	0.87 ± 0.06	0.720	<0.001
L Lateral Temporal	0.86 ± 0.07	0.86 ± 0.06	0.756	<0.001
R Mesial Temporal	0.66 ± 0.09	0.67 ± 0.10	0.651	<0.001
L Mesial Temporal	0.66 ± 0.08	0.66 ± 0.09	0.636	<0.001

Correlation analysis revealed a significant correlation between regional median SUVRs of eFBB and FDG PET in all brain regions (p < 0.001). Likewise, aggregating all the subregions in the whole sample a very strong correlation was revealed (Spearman rho = 0.879).

**Figure 1.**
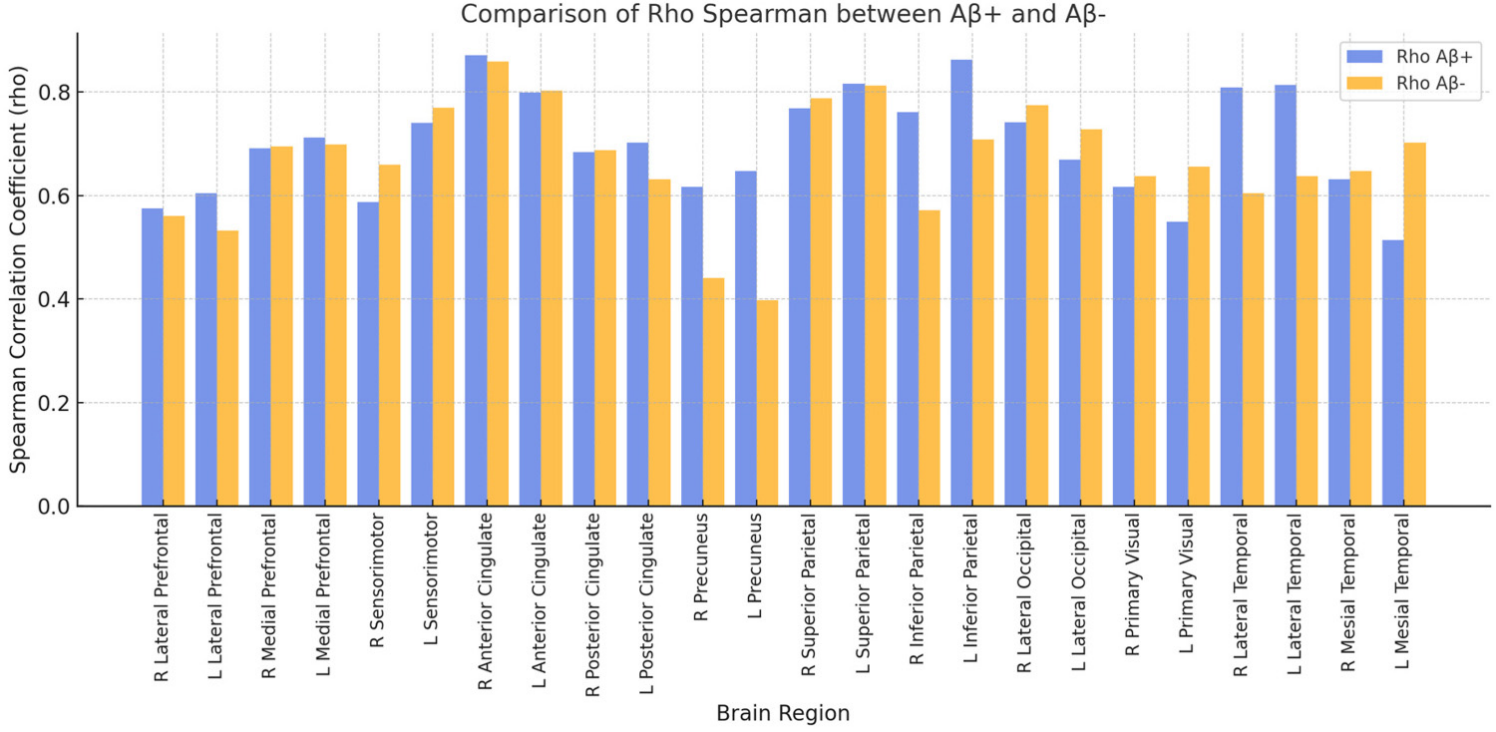
Comparison of Spearman's correlation coefficients (rho) between eFBB and FDG PET in Aβ+ and Aβ− patients. The bar plot illustrates rho values for each brain region, analyzed separately for Aβ+ (blue/ left bar of each pair)) and Aβ− (orange/ right bar of each pair) groups (colors are visible in the online version).

To further investigate whether amyloid status influences eFBB results, we analyzed the correlation between eFBB and FDG PET separately for Aβ+ and Aβ− patients. Spearman's rho values for each brain region are shown in Figure 1. Additionally, Fisher's z-test was conducted to statistically compare these correlations (Table 3).

**Table 3.** Spearman's rank correlation coefficients (rho) for each brain region in Aβ+ and Aβ− patients and Fisher's z-transformation test values.

Brain Region	rho Aβ+	rho Aβ-	Z-score	p
R Lateral Prefrontal	0.575	0.560	0.108	0.914
L Lateral Prefrontal	0.605	0.532	0.526	0.599
R Medial Prefrontal	0.691	0.695	−0.037	0.970
L Medial Prefrontal	0.712	0.699	0.126	0.899
R Sensorimotor	0.587	0.660	−0.583	0.560
L Sensorimotor	0.740	0.770	−0.340	0.733
R Anterior Cingulate	0.871	0.859	0.232	0.816
L Anterior Cingulate	0.799	0.803	−0.054	0.956
R Posterior Cingulate	0.684	0.688	−0.037	0.970
L Posterior Cingulate	0.703	0.631	0.633	0.526
R Precuneus	0.617	0.440	1.206	0.227
L Precuneus	0.647	0.398	1.697	0.089
R Superior Parietal	0.769	0.788	−0.235	0.814
L Superior Parietal	0.816	0.812	0.058	0.954
R Inferior Parietal	0.761	0.572	1.694	0.090
L Inferior Parietal	0.863	0.708	2.052	0.040
R Lateral Occipital	0.741	0.775	−0.389	0.696
L Lateral Occipital	0.669	0.728	−0.562	0.574
R Primary Visual	0.617	0.638	−0.169	0.866
L Primary Visual	0.550	0.656	−0.814	0.415
R Lateral Temporal	0.809	0.604	2.066	0.038
L Lateral Temporal	0.814	0.638	1.868	0.061
R Mesial Temporal	0.631	0.647	−0.132	0.895
L Mesial Temporal	0.514	0.703	−1.484	0.137

Only 2 out of 24 regions showed statistically significant differences (p < 0.05), which may be due to statistical variability rather than a true biological effect, while all other regions demonstrated no significant differences (p ≥ 0.05). These findings indicate that amyloid status has minimal impact on eFBB results.

**Figure 2.**
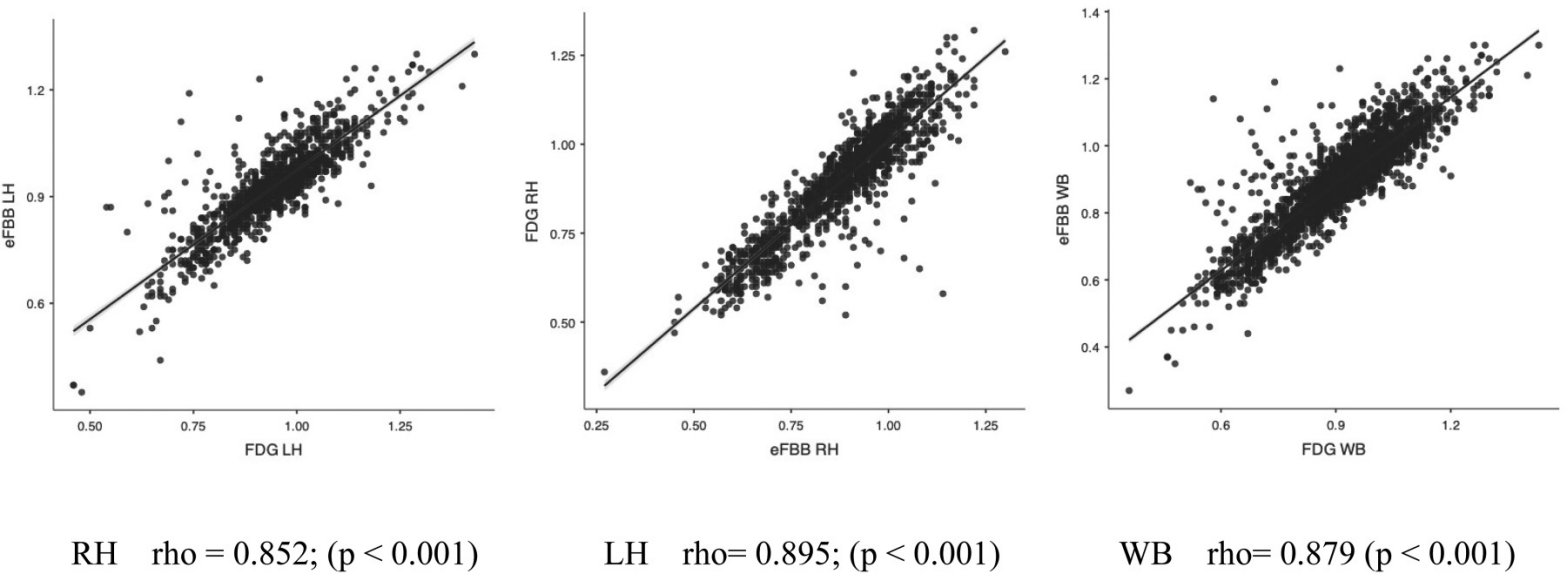
Correlation between eFBB SUVR and FDG SUVR. Scatterplots illustrating the association between eFBB SUVR (y-axis) in cortical subregional VOIs and the corresponding FDG SUVR (x-axis). The results are displayed separately for the right and left hemispheres (RH and LH), as well as for the entire set of subregions (WB). Linear regression lines are depicted in black, with standard errors (SE) indicated in blue. R and P values are provided in the lower section of the plots (colors are visible in the online version).

Furthermore, separating into right hemisphere (RH) and left hemisphere (LH), a very strong correlation was also found between both procedures (RH rho = 0.895 and LH rho = 0.852 for the LH) (Figure 2).

In addition, a strong or very strong correlation was observed in all the VOIs studied. The highest correlation was observed in the right anterior cingulate (rho = 0.857), and the lowest correlation was observed in the left lateral prefrontal cortex (rho = 0.570).

Additionally, we performed a subgroup analysis based on clinical diagnosis (MCI and dementia) and amyloid status (Aβ+ and Aβ-) to assess whether the relationship between eFBB PET and FDG PET varied across subgroups. Spearman's correlation coefficients were as follows: MCI Aβ+ (rho = 0.719), MCI Aβ- (rho = 0.523), Dementia Aβ+ (rho = 0.701), and Dementia Aβ- (rho = 0.719). Comparisons using Fisher's z-transformation test revealed no statistically significant differences between subgroups (all p > 0.30), suggesting that the relationship between eFBB PET and FDG PET remains stable regardless of clinical diagnosis or amyloid status.

### Visual assessment of eFBB and FDG PET images for clinical diagnosis

After blind assessment of both eFBB and FDG PET scans by two readers for the four possible diagnoses (Normal; Possible AD; Probably AD and Other), a concordance correlation coefficient of 0.89 was achieved.

In the intra-observer evaluation of each scan, Reader 1 achieved an agreement of 87.5% and a Cohen's Kappa value of 0.78 (k = 0.78).^69^ Reader 2 achieved an agreement of 86.4% and the same Cohen's Kappa value of 0.78. Further details and specific results are provided in Table 4. Additionally, inter-observer agreement values were obtained for eFBB, with 74.8% agreement and a k = 0.55, and for FDG, with 68.9% agreement and a k = 0.51.

**Table 4.** Contingency tables reporting frequency of different hypometabolism and hypoperfusion patterns for each two readers.

eFBB PET / FDG PET	0	1	2	3	Total
Reader 1					
0	56 (54.4%)	9 (8.7%)	0	0	65 (63.1%)
1	1 (1.0%)	21 (20.4%)	2 (1.9%)	1 (1.0%)	25 (24.3%)
2	0	0	8 (7.8%)	0	8 (7.8%)
3	0	0	0	5 (4.9%)	5 (4.9%)
Total	57 (55.3%)	30 (29.1%)	10 (9.7%)	6 (5.8%)	103 (100%)
eFBB PET / FDG PET	0	1	2	3	Total
Reader 2					
0	45 (43.7%)	12 (11.7%)	0	1 (1.0%)	58 (56.3%)
1	0	27 (26.2%)	1 (1.0%)	0	28 (27.2%)
2	0	0	13 (12.6%)	0	13 (12.6%)
3	0	0	0	4 (3.9%)	4 (3.9%)
Total	45 (43.7%)	39 (37.9%)	14 (13.6%)	5 (4.9%)	103 (100%)

0 = Normal; 1 = Possible AD; 2 = Probably AD; 3 = Other (neurodegenerative or non-neurodegenerative).

From a total of 206 eFBB and FDG PET scans and corresponding comparisons performed by 2 nuclear medicine physicians experts, 27 (13.1%) cases of diagnosis mismatch were found. In 22/27 cases, readers ascertained eFBB images to be *normal* but made a diagnosis of *possible AD* (21 cases) or *other* neurological pathology (1 case) on the FDG images. In the remaining 5/27 cases, on the eFBB images all five cases were considered *possible AD*, while 3 were considered *probable AD*, one *other*, and one *normal* on the FDG PET.

### Single-subject voxel-wise analysis from binarized SPM t-maps

**Figure 3.**
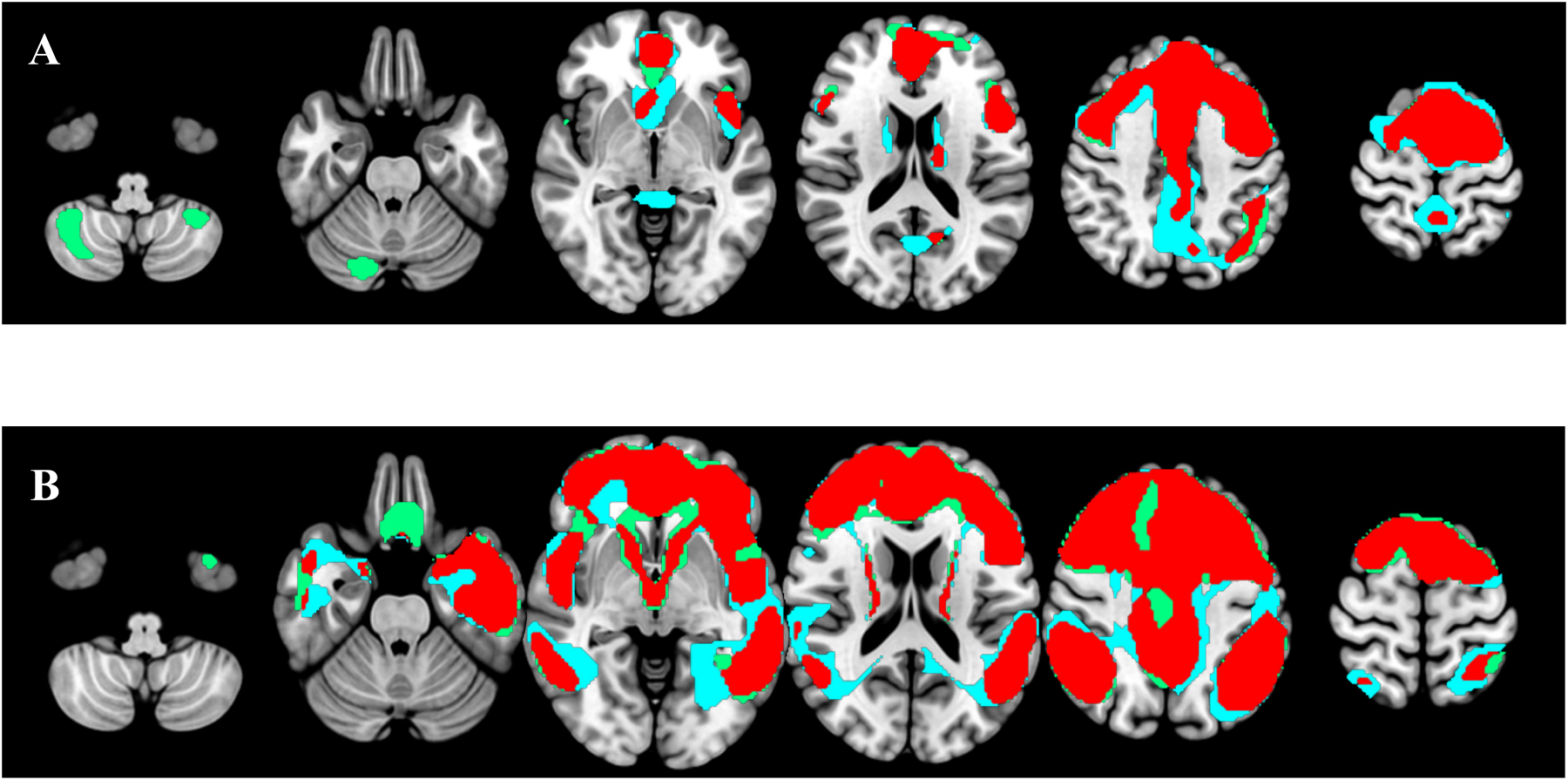
Hypoperfusion and hypometabolic patterns and their overlap at the single-subject level. In blue, eFBB SPM binarized t-map. In green, FDG SPM binarized t-map, and in red, the overlap of both images. The standard Montreal Neurological Institute template was used to overlay the hypometabolism and hypoperfusion maps, along with their overlap. A) Patient with suspected AD and negative lFBB. Hypoperfusion and hypometabolic patterns are not indicative of neurodegeneration in AD. Dice index 0.76. B). Patient with suspected Alzheimer's disease and positive lFBB. Hypoperfusion and hypometabolic patterns are compatible with AD. Dice index 0.79 (colors are visible in the online version).

Stratifying the results into MCI and Dementia categories, in accordance with clinical suspicion, we obtain the following outcomes:

MCI: Dice score indicated a moderate-to-good degree (0. 4< Dice < 0.8) of overlap in most MCI cases (33/44–75%) with Dice average = 0.65 and 0.55 for Aβ+ MCI and Aβ- MCI respectively. However, within the MCI group there was variability, with subjects presenting limited overlap between eFBB and FDG PET, one MCI subject presented a Dice of 0.17 (Aβ- and both, eFBB and FDG PET classified as normal) and 7 MCI subjects presented a poor-to-fair agreement with a Dice score lower than 0.4 (5 Aβ- and 2 Aβ+) all of them also classified as normal on both, eFBB and FDG PET images.
Dementia: Dice score indicated a moderate-to-good degree (0.4 < Dice < 0.8) of overlap in most Dementia cases (51/59–86.4%) with Dice average = 0.69 and 0.67 for Aβ+ Dementia and Aβ- Dementia respectively. In this group, we did not find any patients with a Dice coefficient categorized as poor (less than 0.2), and only 4 out of 59 had a coefficient below 0.4 (moderate). Among these, 3 were Aβ- (2 normal for both eFBB and FDG PET and 1 possible AD on eFBB and probable AD on FDG) and 1 was Aβ+ (normal for both eFBB and FDG PET) (Figure 3).

Upon conducting a paired samples t-test, no significant differences were observed between the Aβ+ and Aβ- subgroups (p = 0.252), nor between the MCI and Dementia subgroups (p = 0.066).

### Discriminative performance between eFBB and FDG PET

**Figure 4.**
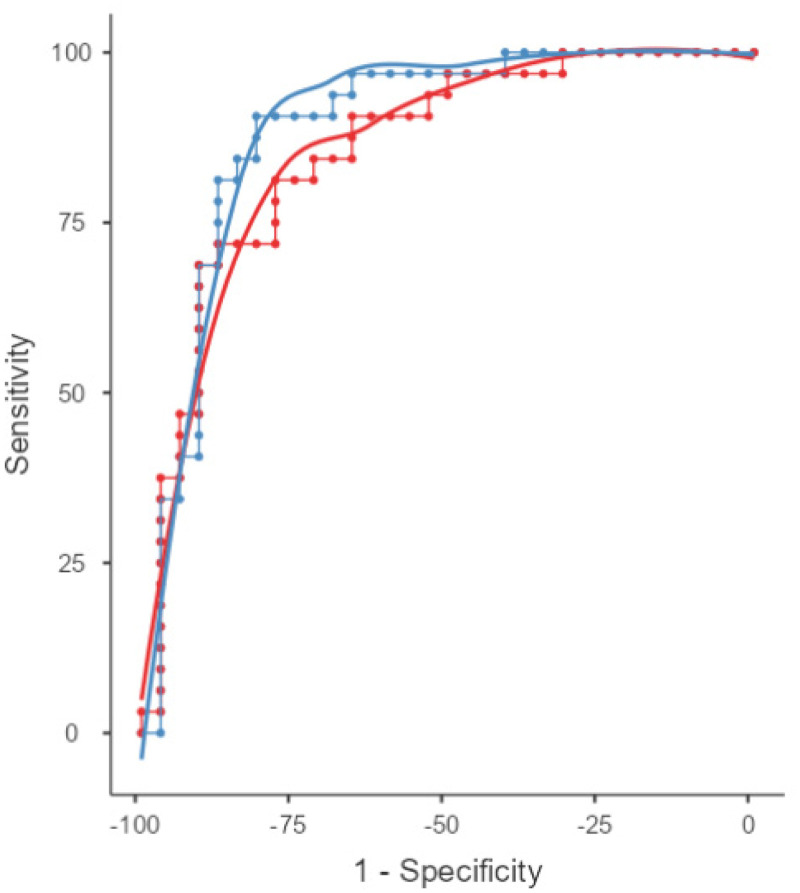
Discriminative performance of eFBB and FDG PET for classification. ROC curves showing diagnostic performance of FDG PET and eFBB SUVr AD related meta-ROI in distinguishing AD from HC. AUCs for eFBB PET is shown in red and for FDG PET is shown in blue. The AUC for FDG PET was 0.888, while the AUC for eFBB was 0.856 (slightly higher) (colors are visible in the online version).

To assess the discriminative power of FDG PET and eFBB-PET in identifying AD, we analyzed the subgroup of Aβ+ patients. SUVRs was computed for a meta-ROI previously described, and the same procedure was applied to the subgroup of HC. The AUC was 0.888 (SD = 0.045) for FDG PET and 0.856 (SD = 0.048) for eFBB PET, both significantly greater than chance (p < 0.001) (Figure 4). However, the pairwise DeLong test revealed no statistically significant difference in discriminative ability between the two modalities (AUC difference = 0.031, 95% CI: −0.034 to 0.096, p = 0.345). These findings suggest that both FDG PET and eFBB PET exhibit high diagnostic accuracy for AD, with no significant advantage of one modality over the other.

## Discussion

This study investigates the comparability between early-phase amyloid PET imaging using FBB, which assesses cerebral perfusion, and cerebral metabolism evaluated with FDG PET. Analyses were conducted at both group and single-subject levels, with a primary focus on semiquantitative and voxel-wise analyses. Furthermore, we assessed the diagnostic accuracy of these imaging modalities in comparison to the definitive diagnosis established by late-phase amyloid PET imaging (AD and non-AD), using the CL, with a threshold of CL ≥ 35 as the standard of truth for confirming AD. While this approach provides a biologically validated threshold for amyloid deposition, it is acknowledged that amyloid positivity alone may not fully capture the complexity of AD pathophysiology. These findings underline the importance of integrating amyloid PET results with clinical and metabolic imaging data to refine diagnostic accuracy and better capture the heterogeneity of neurodegenerative conditions.

While previous studies have conducted semiquantitative, qualitative, and voxel-wise comparative analyses of early-phase amyloid PET and FDG PET, our study represents the largest cohort using FBB and one of the most substantial cohorts for a single radiotracer, further reinforcing the utility of this radiopharmaceutical for both early- and late-phase imaging.^15–27^ Additionally, to date, our study is the only one that has evaluated the comparability of early-phase FBB PET and FDG PET at the single-patient level, providing unique insights into its potential clinical applicability. Notably, only one other published study, which utilized FMM and FPB, has reported similarly robust results, further underscoring the growing evidence supporting early-phase imaging as a valuable biomarker in neurodegenerative diseases.^27^

At the group level, semiquantitative analysis of cortical surface VOIs revealed a strong to very strong correlation between eFBB and FDG PET images in terms of subregional mean SUVR values across all brain regions. This finding aligns with previous studies that used FBB as a single radiotracer or in combination with other amyloid PET tracers.^15–17,21^

To further investigate whether amyloid status influences this correlation, we performed a subgroup analysis separating Aβ+ and Aβ− patients. The results showed no statistically significant differences in 22 out of 24 brain regions (p ≥ 0.05), indicating that amyloid status does not substantially affect eFBB PET measurements. These results suggest that eFBB PET provides stable perfusion information regardless of amyloid status. The two regions that did show significant differences (p < 0.05) may be due to statistical variability rather than a true biological effect, as no clear pattern was observed.

Additionally, we conducted a subgroup analysis based on clinical diagnosis (MCI versus dementia) and amyloid status (Aβ+ versus Aβ−) to assess whether disease stage further influenced the relationship between eFBB PET and FDG PET. We observed no significant differences between subgroups (all p > 0.30), reinforcing the consistency of eFBB PET correlations across different disease stages and amyloid profiles. These findings support the robustness of eFBB PET as a surrogate biomarker for neurodegeneration, independent of clinical status.

Our findings are comparable to those of Son et al., with a similarly strong to very strong correlation, though our cohort was significantly larger (103 versus 40 patients) and included a greater number of subregions (24 versus 8).^15^ Additionally, when grouping subregions into whole brain or hemispheres, we observed similar correlation values, with a correlation coefficient (r) of 0.82 for the whole brain (Spearman's rho = 0.89 in our study), and r = 0.84 and r = 0.79 for the right hemisphere (RH) and left hemisphere (LH), respectively (rho = 0.85 and rho = 0.89 in our study). Given the non-normal distribution of SUVR values, we used Spearman's rho for the correlation analysis. Notably, FDG PET values across the whole brain were significantly higher than those of eFBB, except in the bilateral occipital region, which may be attributed to sample size and lower variability in this region.

Compared to Tiepolt et al., the correlation coefficients in our study were slightly higher.^16^ Their study, which examined a mixed cohort of early ^11^C-PIB and eFBB scans, reported subregional correlations ranging from r = 0.61 to r = 0.79 (using the cerebellum as the reference region). The lower correlations in their study may be attributed to their choice of reference region (cerebellum) as opposed to the general linear model (GLM) used in our study, as well as their smaller sample size and the use of different radiopharmaceuticals.

Similarly, Seiffert et al. reported comparable correlation results between eFBB and FDG PET, with a mean correlation coefficient of r = 0.77.^17^ However, their findings are less comparable due to their smaller cohort (6 patients) and a 1-min acquisition time for eFBB PET. Daerr et al. also demonstrated strong correlation values for all cortical subregions (12 subregions), as well as for the global RH and LH (r = 0.86 and r = 0.87, respectively), which are comparable to our values, though their cohort size was smaller (33 patients).^21^

Beyond the VOI-based semiquantitative analysis, our study also aimed to assess the clinical utility of FBB PET through visual interpretation of eFBB images. Two independent readers reviewed both eFBB and FDG PET images, classifying them into four diagnostic categories (Normal, possible AD, probable AD, or other). Our results showed a very strong visual correlation between eFBB and FDG PET images (r = 0.89). Intra-observer agreement values were k = 0.78 for both readers, while inter-observer agreement was k = 0.55 for eFBB and k = 0.51 for FDG PET. These findings are consistent with those of Daerr et al., who reported a mean intra-observer agreement of k = 0.87, 0.79, and 0.79 across three independent readers.^21^ However, our study's slightly lower intra-observer agreement may reflect differences in clinical diagnoses, adding complexity and subjectivity when distinguishing possible from probable AD. Sample size differences may also account for these variations, as highlighted by the results from our semiquantitative analysis.

When comparing our results with those of Son et al., we found that while they also employed three readers and four diagnostic categories (Normal, AD, FTLD, or non-AD/non-FTLD), their mean correlation (r = 0.88) and intra-observer agreement values (k = 0.83, 0.82, 0.81) were similar to ours.^15^ However, their slightly higher intra-observer agreement could be influenced by their smaller sample size and different diagnostic criteria. Their mismatch rate (15.8%) was also higher than ours (13.1%), with most discrepancies occurring between normal eFBB and AD in FDG PET.

Boccalini et al. reported similar results despite using different amyloid PET tracers (FPB and FMM) and visual evaluation by a single reader.^27^ Their study, involving a cohort of 166 patients, reported a 13% mismatch between early-phase amyloid PET and FDG PET, with six diagnostic categories.

We also evaluated single-subject voxel-wise analyses from binarized SPM t-maps. Stratifying results by clinical suspicion of MCI or dementia, we obtained moderate-to-good spatial overlap between eFBB and FDG PET images, with a mean Dice coefficient of 0.64 across the entire sample. When disaggregated by clinical suspicion and late-FBB status (Aβ+ or Aβ-), Dice coefficients ranged from 0.55 to 0.69, consistent with or slightly higher than the 0.53 reported by Boccalini et al.^27^ The moderate-to-good overlap in our study reflects the ability of eFBB to identify neurodegenerative patterns similar to those seen in FDG PET, despite differences in the biological processes measured by each modality.^8,23^

Overall, both eFBB and FDG PET demonstrated good diagnostic performance in distinguishing AD from non-AD patients, with weak to strong discriminatory values for both modalities. Although FDG PET performed slightly better in ROC analyses, this difference did not reach statistical significance, as confirmed by the DeLong test. This finding aligns with the results reported previously, who also found no significant differences between eFBB and FDG PET in discriminatory power based on SUVRs of meta-ROIs.^27^

Patients included in this study were evaluated in a routine clinical setting, reflecting a diverse population referred by expert dementia neurologists. Although this may introduce variability in the results, we believe the cohort is sufficiently large and representative of a tertiary memory clinic population.

A limitation of this study is its single-center, cross-sectional design, which may limit the generalizability of the findings. Nevertheless, this is the first study, to our knowledge, to assess the classification performance of early-phase amyloid PET with FBB at the single-subject level using voxel-based analysis. Furthermore, this is one of the largest cohorts evaluating the comparability of eFBB and FDG PET at both group and individual levels. The high correlation between eFBB and FDG PET images, as demonstrated by semiquantitative analysis, voxel-wise comparison, and visual interpretation, supports the use of eFBB as a reliable alternative to FDG PET in clinical practice.

### Conclusion

Based on our results, eFBB appears to be a reliable surrogate biomarker for FDG PET in the diagnostic process of neurodegenerative dementias. This supports the inclusion of dual-phase amyloid PET with FBB in routine clinical practice, enabling the simultaneous assessment of neurodegeneration and amyloid status with a single radiopharmaceutical in one diagnostic procedure.
